# On the Purity and Use of Chloroform

**Published:** 1851-08

**Authors:** 


### SELECTIONS

FROM

### FOREIGN AND HOME MEDICAL JOURNALS.

---

*Chloroform.*—In the Transactions of the New York Academy of Medicine, recently published, we find an excellent paper on Chloroform, by Dr. J. T. Metcalfe, from which we make the following quotations:

With a view to ascertain with how much safety we may venture to administer chloroform, such as we find it in the shops, I have obtained about twenty specimens from different apothecaries, and have tested them; first, by thc color, secondly, by the odor, thirdly; by the specific gravity, fourthly, by reaction on litmus paper, fifthly, by agitation with chemically pure sulphuric acid. As a general rule, the specimens examined have been colorless. In one or two, however, a slight tinge of yellow has been noticed. This chloroform has possessed a strong unpleasant odor, and has excited irritation of the air passages, headache, and nausea, when inhaled. I should consider its use as highly dangerous. When kept in glass stoppered bottles, this liquid deposits around the stopper a yellowish, disagreeable pungent substance, of the consistence of oil—perhaps some of the chlorinated oils of Soubeiran and Mialhe. In the sample presented, the specific gravity is high—reaching 1492. On allowing it to drop on a sheet of litmus paper, a deep red mark is left after avaporation, giving evidence of a considerable quantity of free acid. On agitation with one-third its volume of pure sulphuric acid, a faint discoloration of the latter is observable, due to the carbonization of some of the foreign bodies in the chloroform.

In another specimen, possessing the disagreeable odor mentioned, in which the specific gravity was also above 1490, prolonged contact with chemically pure oil of vitriol produced only the faintest shade of darkening. This chloroform, however, gave an acid reaction with litmus paper; and after standing for some time, stained the cork of the phial a brownish yellow color.

At the suggestion of Professor Wolcott Gibbs, of the Free Academy, a specimen offering these characters was treated with the peroxide of lead. By this the disagreeable smell, previously existing, was completly removed. It was attributed by Prof. Gibbs to the presence of sulphurous acid, probably mixed with some of the chlorinated oils of Soubeiran, or with the vapor of chloroform itself.*

It has been stated, as a general rule, that the specific, gravity of chloroform may be taken as a test of its purity. This was lately mentioned to me by the clerk of one of our best apothecaries, as a proof of the excellent quality of the article he had sold me. The facts above stated, as well as Professor Gregory's tables, go to prove the error of this belief, and should admonish us to be careful in examining all specimens of the drug before giving it by inhalation.

The great majority of the samples examined were from the manufactory of Farr, Power, and Weightman of Philadelphia, and in almost every instance, were found to be very nearly pure. The specific gravity of no specimen examined was so high as 1500, nor was there any below 1484. This, in the early history of chloroform, was considered to indicate a very fair quality of the article.

With reference to the odor of such chloroform as came under my observation, it was rare to meet with any in

---

* For the benefit of those who may wish to test the purity of chloroform, after Gregory's process, I would state, that in order to do so satisfactorily, it is necessary to have *chemically pure* sulphuric acid. This is perfectly colorless, and enables us by a faint brown tinge, to detect a very small amount of foreign bodies.

which the fragrant, peculiarly pleasant smell of the pure liquid existed. It would be impossible in words to explain in what the difference consisted, but I have practically found it an easy matter to tell by the "bouquet," whether any of those substances were present which have appeared to cause headache or bronchial irritation. By comparing the two specimens marked I and G the difference will be readily appreciated, the latter being perfectly pure chloroform, rendered such by the process of Gregory. The former was presenfed me by a gentleman who had attempted its use in a surgical operation, It caused such violent laryngeal irritation, however, as to oblige him immediately to desist from its use.

This sample is similar in odor and other respects to another specimen obtained from a dentist who seemed to consider it of good quality. His assistant has since informed me that the whole bottle had been returned to the apothecary from whom it had been purchased, on account of its having nearly strangled a lady, to whom it had been administered for the extraction of a tooth.

Another method of testing the purity of chloroform, by the sense of smell, consists in allowing a little to evaporate from a handkerchief. When the impurities alluded to exist, they are rendered more strikingly manifest by the odor remaining after evaporation.*

---

* Since writing the above, I have seen a communication from Dr. Christison to the editor of the Monthly Journal of the Medical Sciences, in which he speaks of the chloroform purified by chemists, according to Prof. Gregory's process, as having undergone decomposition after having been kept for some weeks. One specimen, however, thus prepared by Prof. G. had remained unaltered for six months.

I have proved that *prolonged* contact with concentrated sulphuric acid may effect decomposition; but if only sufficient agitation and admixture be employed to get rid of the carbonizable foreign matter, I feel confident that subsequent decomposition would not occur.

I would refer for proof of what I have stated to the specimen marked G, purified, as above described, more than five weeks ago. It is perfectly pure, and has been used several times lately by myself, always with the most satisfactory results.

We shall probably soon learn from the Edinburgh professor of chemistry, the cause of the difficulty complained of, and the manner in which it may be obviated.

*Precautions necessary in Administration.*—In the exhibition of anæsthetics for surgical operations, I consider it in the highest degree essential, that the operator should select some one whose duty it should be to give his *undivided attention* to the action of the vapor, and thus by carefully observing the approach of any unpleasant symptoms, to take such measures as might be necessary to obviate them.

It gives full employment to one person to attend to the proper amount of vapor administered, the state of the respiration, of the pulse, and the general condition of the patient. No one who may be led to watch closely the steps of the operation, is proper to manage the anæsthetic apparatus. I feel confident, that several unfortunate results have been due to a neglect of this precaution.

Having attended to the purity of the article, the first step should be to ascertain, as nearly as possible, the condition of the viscera. I should more especially direct attention to the state of the heart, as regards valvular disease, or the probable existence of fatty degeneration. In my own mind, the death of Mr. Badger, in England, was clearly attributable to the ease with which paralysis of the heart, due to this pathological condition, was effected. Fatty degeneration of the heart has also been recorded, in the post-mortem examination of several other fatal cases.

The existence of pulmonary emphysema, or of tubercles unless in a very advanced stage, I should not consider to contra-indicate the use of ether or chloroform. I have administered the latter with the happiest effects to patients laboring under these diseases; and in the case of emphysema, it has rather seemed to increase than diminish the facility with which respiration was carried on. To epileptic patients requiring a surgical operation, the same remarks would apply. I have never yet exhibited it to one, preparatory to the use of the knife, but have several times administered it, to cut short or prevent a paroxysm of the disease. In fine, with regard to chloroform, I may use the words of Dr. Haywood, of Boston, in writing of sulphuric ether: "I hardly know a state of the system, in which I should be deterred from using it, if necessary for a surgical operation, or for the relief of severe pain."

I consider the *position* of the patient a matter of great importance. It should, if possible, be free from constraint, and as nearly horizontal as may be. By attention to this we obviate the tendency to syncope, and allow the respiratory movements to be performed more freely and more easily than when the patient is upright. I have always observed this precaution when rendering persons insensible to pain for surgical or dental operations.

Thus far, in the obstetrical use of chloroform, I have not found a single recorded instance in which death has ensued from its administration. This has been attributed to the comparatively slight degree of anæsthesia produced in these cases. For myself, whilst I feel that this may perhaps have been true in a few instances, I cannot think that the *degree of narcotism* is sufficient to explain the exemption which has so remarkably characterized its use in parturition. In several of the fatal administrations, previously mentioned, the quantity used has been exceedingly small, and the time so short as to prove that complete anæsthesia could not have been produced—in the ordinary way. In my opinion, the horizontal position has been the great cause of immunity from fatal results in obstetrics.

In cases where death has been caused by chloroform, it has been impossible, in every instance, to ascertain the position of the patient. It is extremely probable, however, that in twelve out of the eighteen mentioned, the persons were *sitting* at the time of inhalation.

Occassionally, patients are met with who have to be instructed in the manner of inhaling. If left to themselves, they fail to take the vapor into the lungs in sufficient quantity to produce the desired effect, and we have those unpleasant feelings which almost always accompany an inefficient administration of the anæsthetic. In this way I have once or twice known a person resist the narcotic influence for half an hour before becoming insensible.

In surgery, it is frequently advisable, in order to avoid a shock in the way of mental impression, that the anæsthetic state should not be produced in the appartment where the operation is to be performed. It is also desirable that the patient, when practicable, should after the operation be removed to an adjoining room, in order that, on recovering his senses, no shock may be effected by the necessity of seeing the bloody hands or instruments of the operator, or the portion that may have been removed by the knife. I am cognizant of one instance in which alarming syncope was induced by the patient suddenly beholding his amputated limb on recovering consciousness.

It is prudent to observe the precaution already pointed out by writers, with reference to the condition of the stomach. This should be empty at the time of the operation, as nausea is thus much less apt to be induced, and the surgeon is not annoyed by the vomiting of the patient, and consequent interruption of his work.

I have never known any serious symptoms as a consequence of vomiting from chloroform, and must refer to the account of *possibility*, the dangers which have been insisted on as liable to ensue from closure of the glottis by the matters ejected from the stomach.

*Mode of Administration.*—With regard to the manner in which chloroform should be administered, my experience has led me to adopt the plan of giving the vapor slowly from a handkerchief, the latter never being allowed to come in contact with the face, in order to insure a free admixture of atmospheric air at each inspiration. In some of Professor Simpson's papers, the contrary course was advised, viz: to give at once an overwhelming dose of the vapor, so as to avoid the stage of muscular excitement.

There can be no doubt that this plan is more dangerous than the one above mentioned. In many cases, without doubt, etherization may be safely effected by this method, but in constitutions peculiarly susceptible to the influence of the vapor (as in the case of Mr. Badger,) it should not be left for the practitioner to confess that he has neglected any one of the precautions which should be observed in the exhibition of so potent an agent. My own experience has furnished several instances in which the truth of these remarks has been made apparent. Bronchial irritation, cough, and congestion of the face, which have followed the too rapid filling of the lungs with the unmixed vapor, have all disappeared, and the anæsthetic state been kindly produced when the precaution mentioned has been attended to.

Dr. Snow, of London, still continues to advocate the use of the inhaler, and attributes many of the fatal cases to using the handkerchief. In Scotland, however, the latter article is, I think, exclusively employed, and the great immunity from fatal consequences which has hitherto attended its use, would seem to corroberate the opinion already expressed as to its superiority, or at least to its possessing equal merit with the inhaler.

I have spoken of the necessity of paying strict attention to the state of the respiratory function during the inhalation. In proportion as the system comes more profoundly under the narcotic influence, are the voluntary muscles of respiration paralysed, until when deep anæsthesia has been effected, we find that the abdominal muscles, diaphragm, and the natural elasticity of the lungs, are the exclusive agents by which breathing is carried on. When this kind of respiration is observed, I have always made it a rule (if there were any considerable degree of dyspnea) to allow the inspiration of air alone, until the difficult breathing had been removed.

As has been generally recommended by those who have practiced the administration of chlooriorm or ether for surgical purposes, it may be ascertained when a patient is in a proper condition for the operation, by watching carefully for the production of voluntary muscular relaxation. The occurrence of stertorous respirations is always a sign of deep narcotism, and should warn us to suspend the anesthetic until the stertor ceases.

*Means to be Used where Anesthesia is carried too far.*—What shall be done in instances where animation is suspended by an overdose of the chloroform? This is a most important question, but one which I think can be much more satisfactorily answerd now than would have been the case a year ago.

I am able to speak of four cases in which after cessation of the breathing, circulating, and sensitive functions—in fact after apparent death, life has been restored by a prompt resort to what should always be our first recourse, viz: *properly performed artificial respiration.*

Two cases in which this means was successful in restoring suspended animation from chloroform vapor, were recorded in a letter from Mr. Ricord, contained in the September number of the *Union Médicale*, for 1849.

In the May (1850) number of the London Lancet, was the report of another instance in which life was recalled, by almost immediately resorting to artificial respiration, after seeing the inefficiency of cold air and ammonia.

The fourth case occurred to myself, and as it may be useful in impressing what I conceive to be the great value of this course of treatment, I have thought it proper to give the circumstances in detail. They were as follows :—

On the 1st of March, 1850, I was requested by Dr. Delafield, of this city, to administer chloroform to a man fifty years of age, for the purpose of having extirpation of the left eye-ball performed, on account of fungus of the part. The man had previously been in general good health, and at the time of the operation was stout and robust. He was carefully examined to ascertain if there was any visceral disease, but no indication of its existence was detected. He had been accustomed to indulge freely in the use of intoxicating drinks.

As I have frequently remarked to be the case in intemperate persons, he was long in coming under the influence of the chloroform vapor, which was administered from a handkerchief, with the precautions hitherto mentioned. His position was recumbent, on the back. In the course of inhalation, before complete anesthesia was effected, there was a considerable amount of convulsive muscular action and incoherent talking.

Seventeen minutes after the vapor was applied, there was slight stertorous respiration, and complete muscular relaxation. He was also insensible to tolerably hard pinching.*

The operation (not requiring more than a minute of time) was then performed by Dr. Delafield, and was immediately followed by a smart gush of arterial blood from

* In this man's case there was the indisposition to respire, before alluded to. It was frequently necessary to speak to him sharply, and to excite inspiration by pressing the walls of the chest during the administration of the chloroform.

the wound. Whilst the incisions were being made, the patient groaned slightly, and made the automatic muscular motions not unfrequently seen, when anesthesia is not carried to its most profound extent.

A few seconds after the globe of the eye had been removed, Dr. Van Buren, whose finger was on the radial pulse, observed that it suddenly ceased to beat. On examining to see if respiration were still going on, this was also found to have stopped. The face previously, somewhat livid, became rapidly blanched and cadaverous, and the hemorrhage instantly ceased.

The true condition of matters was at once appreciated. To all appearances, life was extinct. The window before which the patient's head lay, was thrown open, a towel wet with cold water was slapped in his face, his body was shaken roughly, with the hope that he might be aroused. These efforts were persevered in for about a minute without the slightest success. Not a single sign of animation manifested itself.

Happening at this moment, to remember the two cases in which M. Ricord had been successful in restoring patients under similar circumstances, I at once applied my lips to those of the patient, holding his mouth open with my right hand and closing his nose with the left, and inflated the lungs, slowly and gently, so as to imitate as much as possible a natural inspiration. Dr. Van Buren, by compressing the thorax and pushing up the diaphragm, performed artificial expiration, whilst Dr. Markoe assisted by pressing the thyroid cartilage against the spinal column.

After some fifteen or twenty inspirations thus performed, occupying nearly two minutes, the patient gave a feeble gasp. This was simultaneous with a slight spirt of blood from the artery, furnishing the hemorrhage referred to. Artificial respiration was continued as above described for a minute afterwards, the pulse regaining its strength and fulness, and the face resuming its natural color as we proceeded. The hemorrhage from the wound returned, the breathing became natural, and, in a few minutes, we had the satisfaction of seeing our patient restored to life, and of hearing him speak of the operation.

From this time, no unpleasant symptom showed itself.

His convalescence was as rapid and favorable as the nature of the operation would allow, and he was soon enabled to return to his home, in an adjoining state.

From the results following the measures adopted in this case, in M. Ricord's two cases, and in that of M. Bleeck, there can, in my mind, be no doubt that our first duty in the event of suspended animation, from the use of chloroform, is to proceed *immediately* to the performance of artificial respiration.

In looking over the reports of fatal cases, I have been struck with the fact, that this means has seldom been resorted to, until all others had proved unavailing. The following is a fair illustration of the course usually adopted hitherto. It is taken from an account given of Mrs. Summers's case, the first fatal one in this country. "Ammonia was applied to the nostrils, cold water was dashed in the face, mustard, brandy, etc., applied, but she did not breathe nor exhibit any sign of life, after being placed in the recumbent position."

This case occurred during the early history of chloroform. In it, artificial respiration is not mentioned as having been employed.

In the last case, in which inhalation of chloroform was followed by death—that of Mr. Cock, of Guy's Hospital, the course adopted seems to me to have been very strange, considering what had already been published as to the means of resuscitation. It was as follows:—"Brandy, ether, ammonia, cold air, cold water, shaking, rubbing, electricity, and artificial respiration, for which purpose *the larynx was opened and a tube inserted.* If the artificial respiration, performed properly, had been tried at first, and the brandy used to bring up the rear of this host of remedies, I think the result would, very probably, have been different.

I have thought it proper to insist on the necessity of inflating the lungs, gradually and gently, so as to avoid the possibillity of rupturing the delicate tissues of the air cells. Is it at all improbable that some of the air mentioned as having been found in the veins, on post-mortem examinations, may have been owing to too powerful insufflation, in the attempt to resuscitate?

Theory as well as practice would go to show the uselessness of filling the patient's mouth with brandy, cold water, or anything else, whilst the power of swallowing was abolished. Of no more utility would be the attempt to administer nitrous oxide or oxygen gas, as has been proposed. These can only be introduced into the lungs by inspiration. If the patient could perform this act, there would be no need of the means referred to, as the atmospheric air would answer the desired purpose.

I may mention in this place, that another means, said to have been successful in two cases threatening a fatal termination, consists in thrusting the fore and middle fingers as far as possible down the patient's throat, so as to produce irritation of the larynx. This was proposed and has been put in practice by M. Escalin. The details of the cases I am unable to give.

It would have afforded me pleasure to state to the Academy, the results of my experience in the use of chloroform and ether in the treatment of medical diseases and in obstetrics, but the length to which my paper has already extended, prevents me from more than alluding to this part of the subject at present.

I may mention that I have continued their use in parturition since the first employment of ether in this city, in October, 1847, and have had no cause to regret their administration. My testimony to their happy influence in obstetrical practice would differ in nothing from that of Channing, Simpson, Murphy, and others, whose experience has enabled them to judge fairly of their merits.*

In one case, in which the mixture of ether and chloroform was inhaled, puerperal mania followed parturition. The patient, in this instance, was seen by Dr. Samuel Henry Dickson, and by the late Dr. McDonald, of Sandford Hall, who both expressed themselves of opinion that the attack was in no wise different from those usually seen in this disease. Recovery took place in the course of five or six months. In the somewhat violent paroxysms

---

* I have one patient who has been confined three times whilst in the anesthetic state. On the first occasion ether was used; on the second and third, chloroform was employed. The recoveries were all speedy and uninterrupted. The children all, at present, stout and healthy. The lady in this case, for violent neuralgic pains, during pregnancy, resisting all other remedies, has taken chloroform at least one hundred times. She is now in remarkably good health.

of mania, to which this woman was subject, the tranquillizing effect of chloroform inhalations was beautifully shown. It was repeatedly administered by Dr. Van Buren and myself, and never failed to produce calmness or rest, previous use having been nearly always made of the ordinary narcotic remedies without avail.

In puerperal convulsions, I have known it used six or seven times in New York. In every case except one (in which there was rupture of the uterus) the convulsions were materially shortened, mitigated, or altogether prevented. The cases, with the exception mentioned have all recovered.

In the agonizing pains of dysmenorrhea chloroform has been found of great service. One of my patients, a young unmarried lady, has had it administered at every menstrual period, since its introduction into American practice. It has always afforded great relief to her sufferings, and has never in any way affected her injuriously.

I have several times administered it in violent attacks of colic, when the pain was so excessive as to render it desirable to procure relief as speedily as possibie. In these cases (several of which were even attacks of cholera morbus,) I have found the immediate tranquillizing effects of the chloroform to assist very much the action of the ordinary opiate.

In this country and in Europe, the sedative and narcotic properties of chloroform inhalation have been taken advantage of, in the treatment of delirium tremens. In Bellevue Hospital more than thirty-five cases have been thus treated, and in every instance in which the administration has been steadily and rightly conducted, the result has proved satisfactory. It is necessary with some patients, to remain by their side for an hour or two after sleep has first been procured. As often as a disposition to awaken is shown, the application of the anesthetic should be renewed. In most cases, a long, refreshing, sobering sleep is thus produced, and the patient opens his eyes in a rational condition. In a good many of the Hospital patients alluded to, the ordinary means in general use, had been unsuccessfully tried before resorting to chloroform.

In conclusion, I would state that from the amount of evidence now before us, I do not think we can rightly infer that good chloroform, properly administered, is more dangerous than sulphuric ether. That it is more pleasant, more prompt, and more generally applicable, I have no doubt. With increased experience, I feel disposed to believe that we shall eventually come to understand the indications and contraindications for its use, quite as well as we now understand those of any remedial agent of acknowledged power, in our pharmacopeia.

